# The potential of the serum uric acid to high-density lipoprotein cholesterol ratio as a predictive biomarker of diabetes risk: a study based on NHANES 2005–2018

**DOI:** 10.3389/fendo.2024.1499417

**Published:** 2025-01-23

**Authors:** Jianming Yin, Chuanjie Zheng, Xiaoqian Lin, Chaoqiang Huang, Zhanhui Hu, Shuyuan Lin, Yiqian Qu

**Affiliations:** School of Basic Medical Sciences, Zhejiang Chinese Medical University, Hangzhou, China

**Keywords:** diabetes, serum uric acid, HDL-C, UHR, NHANES

## Abstract

Previous studies have indicated an association between UHR and diabetes risk, but evidence from large-scale and diverse populations remains limited. This study aims to verify UHR’s independent role in diabetes risk prediction in a large sample population and assess its applicability across different populations. We drew upon data from 30,813 participants collected during the 2005–2018 NHANES cycle. The association between UHR and the risk of diabetes was explored using multivariate logistic regression models, with key predictive factors identified through LASSO regression. Model effectiveness was evaluated through receiver operating characteristic (ROC) curves, decision curve analysis (DCA), and calibration metrics. Additionally, restricted cubic spline (RCS) and threshold effect assessments were applied to examine the nonlinear association between UHR and diabetes risk. The results showed that UHR levels were notably elevated in individuals with diabetes when compared to those without diabetes (*p* < 0.001). The occurrence of diabetes showed a marked increase across ascending UHR quartiles (6.63%, 10.88%, 14.15%, 18.02%; *p* < 0.001). Results from multivariate logistic regression indicated that elevated UHR was strongly linked to a heightened risk of diabetes; participants in the highest UHR quartile were found to have nearly four times the risk compared to those in the lowest quartile (OR = 4.063, 95% CI: 3.536–4.669, *p* < 0.001). Subgroup analyses demonstrated that the predictive effect of UHR was more pronounced in females. Key variables selected via LASSO regression improved the model’s performance. Restricted cubic spline (RCS) analysis indicated an inflection point at UHR = 10; beyond this point, diabetes risk accelerated, and when UHR exceeded 18, the risk increased significantly (OR > 1). ROC curve analysis showed the baseline model (M1) had an area under the curve (AUC) of 0.797, while the multivariable model (M4) after LASSO selection had an AUC of 0.789. Decision curve analysis and calibration curves validated the model’s predictive ability and consistency. This study indicates that UHR may be an independent predictor of diabetes risk, showing a positive correlation with diabetes and a more pronounced predictive effect in females.

## 1 Introduction

Diabetes, especially type 2 diabetes (T2DM), has become a major global public health challenge. According to the International Diabetes Federation (IDF), approximately 536.6 million people worldwide were living with diabetes in 2021, and this number is projected to rise to 783.2 million by 2045 (1). Diabetes is a major contributor to multiple serious complications, including cardiovascular disease, kidney failure, retinopathy, and lower extremity amputations (2). Such complications pose a threat to patients’ quality of life and impose a significant strain on healthcare systems worldwide (3).

The key risk factors for diabetes include age, genetics, obesity, lack of physical activity, unhealthy eating patterns, and smoking (4). In addition to these traditional factors, a growing body of research is investigating biomarkers associated with metabolic syndrome, such as elevated uric acid levels, low HDL cholesterol, and insulin resistance (5–7). These metabolic indicators are significant contributors to diabetes and have strong connections with cardiovascular disease development. Therefore, identifying biomarkers that can effectively predict diabetes risk is essential for early intervention and prevention of the disease (8, 9).

In recent years, the serum uric acid to high-density lipoprotein cholesterol ratio (UHR) has attracted considerable interest as an emerging metabolic risk indicator. Uric acid (UA), the end product of purine breakdown, primarily eliminated by the kidneys, can, at elevated levels, suppress nitric oxide production, encourage the proliferation of vascular smooth muscle cells, and cause endothelial dysfunction, thereby speeding up the progression of atherosclerosis and insulin resistance (10, 11). Moreover, reduced low-density lipoprotein cholesterol (LDL-C) levels impair its roles in reverse cholesterol transport, as well as its anti-inflammatory and antioxidant properties, exacerbating lipid metabolic disturbances and further promoting the onset of insulin resistance and metabolic syndrome (12, 13). UHR provides a novel perspective for predicting metabolic disease risk by encapsulating the dual effects of elevated uric acid and reduced HDL-C. Existing research indicates that UHR reflects inflammation and metabolic status, demonstrating outstanding assessment abilities in diseases like Hashimoto’s thyroiditis (14), type 2 diabetes (15) or prediabetes (16), metabolic syndrome (17), coronary artery disease (18), and NAFLD (19). For instance, UHR demonstrates high sensitivity and specificity in forecasting the risk of type 2 diabetes onset, and its mechanism is significantly related to insulin resistance, HbA1c, and FPG levels (20, 21). Research on coronary artery disease indicates that elevated UHR levels are significantly correlated with cardiovascular events, with mechanisms possibly involving accelerated atherosclerosis and endothelial dysfunction (22). Thus, UHR may act as a predictor for diabetes and metabolic syndrome and could also be used for early screening of coronary artery disease and other related cardiovascular event risks.

Although previous research has shown a correlation between UHR and the occurrence of diabetes, along with other metabolic conditions, most are small-scale cross-sectional studies, and findings across different races, ages, and genders are still inconsistent. Consequently, long-term follow-up data from large-scale, diverse populations are lacking to determine the general applicability and predictive performance of UHR as a diabetes risk predictor.

This study uses data from the 2005-2018 NHANES cycles to comprehensively assess the relationship between UHR and diabetes risk, while exploring its predictive ability across different populations. It is hypothesized that UHR may serve as an independent predictor of diabetes and exhibit varying predictive effects across different genders, ages, and racial groups. This study aims to provide new insights into the prevention and risk prediction of diabetes.

## 2 Materials and methods

### 2.1 Data and sample sources

This study utilized NHANES data from the National Center for Health Statistics (NCHS). NHANES is a comprehensive survey designed to collect representative information on the health and nutritional status of the U.S. civilian population, encompassing demographics, socioeconomic status, dietary habits, and health-related issues. To ensure sample diversity, NHANES employed a stratified, multistage probability sampling method to select nationally representative participants. The study protocol was approved by the Ethics Review Committee of the CDC’s NCHS, and all participants provided written informed consent. The data are publicly available at https://www.cdc.gov/nchs/nhanes/.

**Figure 1:**
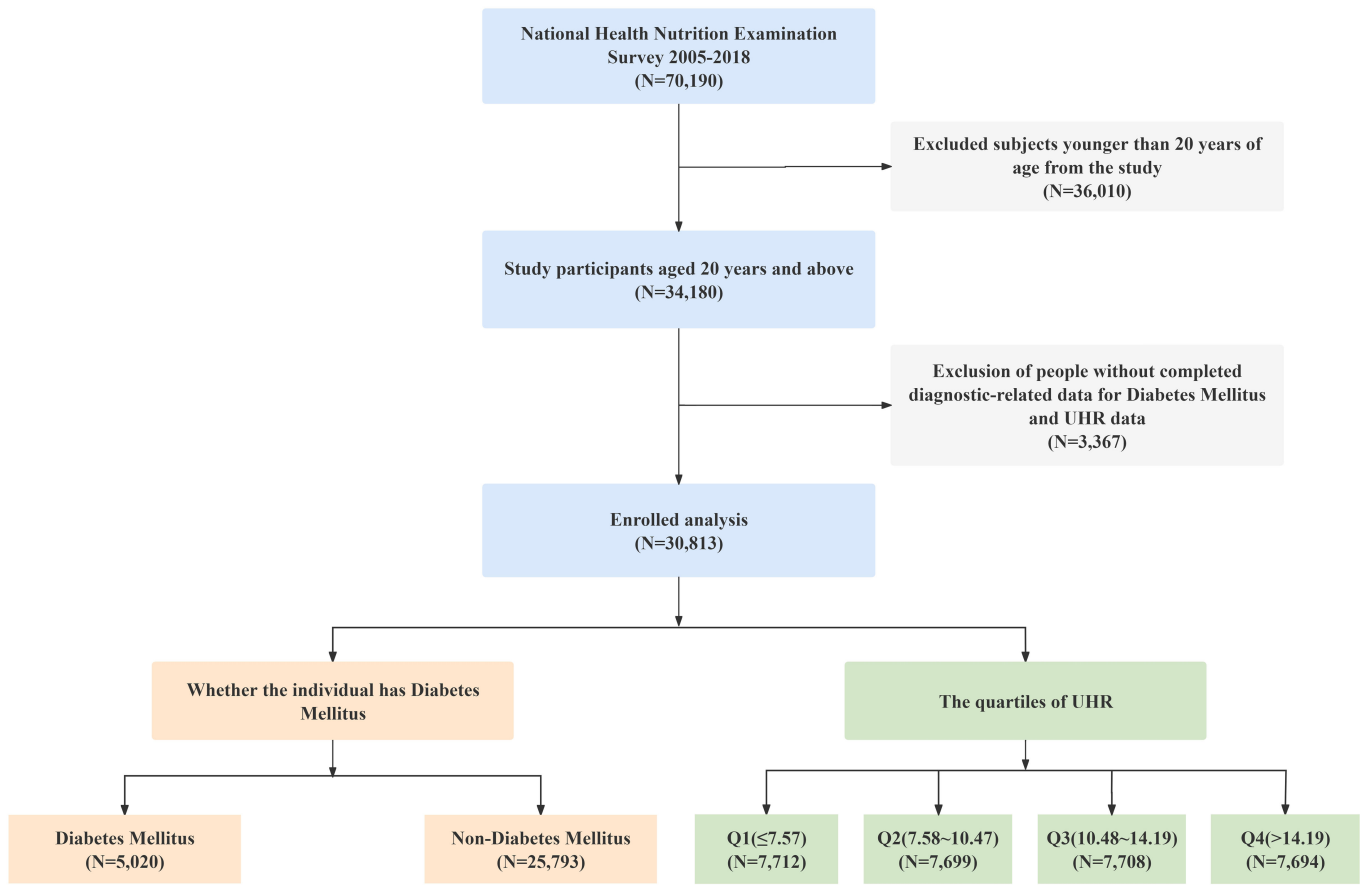
Flow chart.

This study primarily analyzed adult health data from the NHANES 2005–2018 cycles. The original cohort included a total of 70,190 participants. We initially excluded individuals under 20 years of age, followed by those missing diabetes diagnostic indicators and UHR data, ultimately including 30,813 participants, of whom 5,020 were diagnosed with diabetes. The sample selection flowchart is shown in **Figure 1**.

### 2.2 UHR (exposure variable)

The exposure variable, UHR, was calculated using fasting morning blood sample data obtained from the NHANES 2005-2018 database, which provided measurements of uric acid (UA) and high-density lipoprotein cholesterol (HDL-C). In NHANES, HDL-C was measured using direct immunoassay or precipitation methods, while serum uric acid concentrations were assessed via a timed endpoint method. Specifically, UA was measured using a DxC800 automated chemistry analyzer by calculating the change in absorbance of the chromogenic product formed from the reaction between hydrogen peroxide—produced by uricase oxidation of uric acid—and 4-aminoantipyrine (4-AAP) catalyzed by 3,5-dichloro-2-hydroxybenzenesulfonic acid (DCHBS).

The UHR was determined using the following formula: UHR (%) = [UA (mg/dL)/HDL-C (mg/dL)] × 100.

### 2.3 Diabetes (outcome variable)

The outcome variable was diabetes, assessed based on blood glucose parameters and questionnaires, including glycated hemoglobin (HbA1c), fasting plasma glucose (FPG, mmol/L), random plasma glucose (RPG, mmol/L), 2-hour oral glucose tolerance test (OGTT, mmol/L), physician diagnosis, and the use of antidiabetic medications or insulin. Participants were required to fast for 8 to 24 hours prior to laboratory testing; fasting status was confirmed during the morning examination, and laboratory analyses were performed. Because NHANES did not directly provide random plasma glucose data, plasma glucose levels were interpreted in combination with fasting duration: fasting plasma glucose was considered when fasting time was ≥8 hours, and random plasma glucose when fasting time was <8 hours.

Diabetes was defined by meeting any one of the following criteria: (1) HbA1c ≥6.5%; (2) FPG ≥7.0 mmol/L; (3) RPG ≥11.1 mmol/L or OGTT ≥11.1 mmol/L; (4) having been diagnosed with diabetes by a physician; (5) currently taking antidiabetic medications or insulin. Diabetes and non-diabetes were coded as 1 and 0, respectively.

In this study, we prioritized objective criteria (1), (2), and (3) for the diagnosis of diabetes. Only when data for all three objective indicators were missing did we consider criterion (4) (physician-diagnosed diabetes) and criterion (5) (use of antidiabetic medications or insulin). In our analysis, 28 cases were diagnosed based on criterion (4), and none based on criterion (5). Sensitivity analysis indicated that the proportion of self-reported diagnoses was very small, and the potential information bias from this was negligible.

### 2.4 Covariates

Based on existing literature and clinical considerations, we included several confounding factors: sex, age, education level, race/ethnicity, poverty income ratio (PIR), body mass index (BMI), blood pressure (BP), drinking status, smoking status, total cholesterol (TC), triglyceride (TG), LDL-C, HDL-C, non-high-density lipoprotein cholesterol (Non-HDL-C), and UA. Race/ethnicity was classified as: Non-Hispanic Asian, Mexican American, Non-Hispanic White, Non-Hispanic Black, Other Hispanic, and Other/Multiracial. Education levels were divided into three categories: Less than high school, High school or GED, and College or above. PIR was categorized into three groups: <1.30, 1.30-3.49, and ≥3.50. BMI was calculated as weight (kg) divided by height squared (m²). Hypertension was defined as self-reported physician-diagnosed hypertension or current use of antihypertensive medications. Drinking and smoking status were determined based on the questionnaire. All covariates were obtained from the NHANES database.

### 2.5 Missing data handling

In this study, some covariates had missing values, including BMI, TG, SBP, DBP, and LDL. For BMI, TG, SBP, and DBP, the proportion of missing data was less than 10%. We used the k-nearest neighbors (KNN) imputation method to fill in these missing values, with k=5 neighbors, and standardized the relevant variables to ensure the accuracy and stability of the imputation process. For LDL, as the proportion of missing data was greater than or equal to 10%, we chose to retain the variable but exclude the missing values from the analysis, in order to minimize any potential bias arising from the high missing rate. To ensure the robustness of this approach, we also conducted a sensitivity analysis, which demonstrated that neither the imputation nor the exclusion of missing values significantly affected the study’s conclusions, confirming the stability of our findings.

### 2.6 Statistical methods

Data analysis was conducted using DecisionLinnc 1.0 software (23). DecisionLinnc 1.0 is a comprehensive software package that integrates multiple programming languages and is capable of performing various statistical analyses, data processing, and graphical plotting. Given the complex sampling design of the NHANES data, weighted statistical methods were applied.

Participants were divided into two groups based on diabetes status and further categorized into four groups according to UHR quartiles. Continuous variables were assessed using the weighted Student’s t-test or ANOVA, while categorical variables were evaluated with the weighted chi-square test. For continuous variables that did not follow a normal distribution, the weighted Kruskal-Wallis test was used. In the descriptive analysis, continuous variables were expressed as weighted means ± standard deviations, and categorical variables were reported as weighted percentages.

To explore the relationship between UHR and diabetes risk, we initially constructed three multivariate logistic regression models. Before modeling, we evaluated multicollinearity among all covariates using variance inflation factor (VIF) analysis. Model 1 was unadjusted; Model 2 was adjusted for sex, age, and race; and Model 3 included additional adjustments for BMI, PIR, hypertension, smoking and drinking status, SBP, DBP, TC, TG, LDL-C, and non-HDL-C.

Subsequently, we applied LASSO regression to select the variables most significantly associated with diabetes risk, including UHR, age, BMI, sex, and education level, and used these variables to construct an optimized Model 4 to enhance predictive performance and model stability. The nonlinear relationship between UHR and diabetes risk was analyzed using restricted cubic splines (RCS), and the “Intelligent Filtering Restricted Cubic Spline Knot” and “Threshold Effect” techniques were employed to calculate the knot locations and threshold inflection points for each model. The predictive performance of the models was evaluated using ROC curves, decision curve analysis (DCA), and calibration curves, with model stability assessed via the Hosmer-Lemeshow test. A P-value of less than 0.05 in all global statistical tests was considered statistically significant.

Subgroup analysis: To further investigate the association between UHR and diabetes risk across different populations, subgroup analyses were performed on key categorical variables, including sex, race, education level, PIR, hypertension, smoking status, and drinking status. Each subgroup was analyzed using the corresponding multivariate logistic regression model. To control for the risk of Type I error introduced by multiple testing, we applied Bonferroni correction, adjusting the significance level to 0.05/7 = 0.00714. Therefore, in the subgroup analysis, a corrected P-value of less than 0.00714 was considered statistically significant.

## 3 Results

### 3.1 Baseline characteristics comparison between diabetic and non-diabetic groups

This study included a total of 30,813 participants, with a mean age of 47.30 years; 25,793 were non-diabetic subjects and 5,020 were diabetic subjects. Compared with the non-diabetic group, individuals with diabetes had higher age, BMI, SBP, INS, TG, UA, and UHR levels (*p*<0.001), and lower DBP, TC, LDL-C, HDL-C, Non-HDL-C levels (*p*<0.001). Additionally, significant differences were observed across the two groups in terms of gender, ethnicity, educational attainment, PIR, smoking and drinking behaviors, and hypertension prevalence (*p*<0.05). Further details can be found in **Table 1**.

**Table 1:** Baseline characteristics of participants.

Characteristic	Overall	Non-DM	DM	*p*-value
N	30813	25793	5020	
Age (year)	47.30 ± 16.87	45.56 ± 16.53	59.60 ± 13.90	<0.001
BMI (kg/m²)	28.88 ± 6.75	28.36 ± 6.48	32.59 ± 7.40	<0.001
SBP (mmHg)	121.62 ± 15.71	120.65 ± 15.13	128.54 ± 17.89	<0.001
DBP (mmHg)	70.10 ± 11.19	70.32 ± 10.92	68.53 ± 12.85	<0.001
INS (pmol/L)	77.81 ± 66.22	74.13 ± 49.89	103.94 ± 130.67	<0.001
RPG (mmol/L)	6.14 ± 2.42	5.54 ± 1.00	10.00 ± 4.47	<0.001
FPG (mmol/L)	5.88 ± 1.68	5.44 ± 0.54	8.23 ± 3.17	<0.001
OGTT (mmol/L)	6.47 ± 2.69	5.97 ± 1.76	12.75 ± 4.15	<0.001
HbA1c (%)	5.60 ± 0.92	5.39 ± 0.47	7.08 ± 1.68	<0.001
TC (mg/dL)	194.84 ± 41.44	196.10 ± 40.51	185.86 ± 46.58	<0.001
TG (mg/dL)	128.37 ± 76.90	124.98 ± 64.75	152.46 ± 132.21	<0.001
LDL-C (mg/dL)	114.26 ± 25.52	114.97 ± 24.84	109.18 ± 29.46	<0.001
HDL-C (mg/dL)	53.60 ± 16.65	54.31 ± 16.64	48.61 ± 15.84	<0.001
Non-HDL-C (mg/dL)	141.23 ± 41.88	141.79 ± 41.15	137.24 ± 46.51	<0.001
UA (mg/dL)	5.42 ± 1.41	5.37 ± 1.38	5.78 ± 1.56	<0.001
UHR	11.31 ± 5.24	11.06 ± 5.11	13.13 ± 5.74	<0.001
Male (%)	48.26	47.98	50.29	0.027
Race (%)				<0.001
Mexican American	8.52	8.35	9.74	
Other Hispanic	5.32	5.29	5.57	
Non-Hispanic White	68.15	68.82	63.41	
Non-Hispanic Black	10.75	10.33	13.76	
Other Race-Including Muti-Racial	7.25	7.21	7.52	
Education (%)				<0.001
Less than high school	17.09	16.00	24.79	
High school or GED	22.42	22.05	25.03	
College or above	60.50	61.95	50.18	
PIR (%)				<0.001
<1.30	20.10	19.49	24.44	
1.30-3.49	40.05	39.46	44.24	
≥3.50	39.84	41.04	31.33	
Drinking (%)	11.03	10.37	15.67	<0.001
Smoking (%)	45.26	44.52	50.52	<0.001
Hypertension (%)	31.57	26.87	64.97	<0.001

Values for categorical variables are reported as weighted percentages; for continuous variables, as weighted mean ± standard deviation. Statistical analysis included Weighted Student’s t-test for continuous variables and chi-squared test for categorical variables.

BMI, Body mass index; SBP, Systolic Blood Pressure; DBP, Diastolic Blood Pressure; INS, Insulin; RPG, Random plasma glucose; FPG, Fasting plasma glucose; OGTT, Oral glucose tolerance test; HbA1c, Glycohemoglobin; TC, Total Cholesterol; TG, Triglyceride; LDL-C, Low-density lipoprotein cholesterol; HDL-C, High-density lipoprotein; Non-HDL-C, Non-high-density lipoprotein cholesterol; UA, Uric acid; UHR, Uric Acid to High-Density Lipoprotein Cholesterol Ratio; PIR, Poverty Income Ratio.

### 3.2 Association between UHR quartiles and diabetes risk

To explore the relationship between UHR and diabetes risk, participants were categorized into four quartiles (Q1-Q4) based on their UHR values. The results indicated that participants in the higher UHR quartiles had significantly increased BMI, SBP, DBP, INS levels, RPG levels, FPG levels, OGTT levels, HbA1c levels, TG levels, LDL-C levels, Non-HDL-C levels, and UA levels compared to those in the lower UHR quartiles (*p*<0.001), whereas TC and HDL-C levels were significantly decreased (*p*<0.001). Moreover, significant differences were also observed in the distribution of gender, ethnicity, education level, PIR, smoking status, and hypertension prevalence (*p*<0.001). Of particular note, the prevalence of diabetes increased significantly with higher UHR quartiles (6.63% vs. 10.88% vs. 14.15% vs. 18.02%, *p*<0.001). Details are shown in **Table 2**.

**Table 2:** Baseline characteristics by UHR quartiles.

Characteristic	Q1 (≤7.57)	Q2 (7.58, 10.47)	Q3 (10.48, 14.19)	Q4 (>14.19)	*p*-value
N	7712	7699	7708	7694	
Age (year)	47.11 ± 16.72	47.18 ± 17.23	47.64 ± 16.84	47.26 ± 16.69	0.386
BMI (kg/m²)	25.55 ± 5.37	28.23 ± 6.29	29.96 ± 6.76	31.96 ± 6.79	<0.001
SBP (mmHg)	118.88 ± 16.44	120.87 ± 15.44	122.76 ± 15.27	124.13 ± 15.10	<0.001
DBP (mmHg)	68.63 ± 10.57	69.49 ± 10.57	70.74 ± 11.34	71.61 ± 12.02	<0.001
INS (pmol/L)	63.22 ± 42.15	71.79 ± 47.99	79.92 ± 56.78	97.12 ± 98.71	<0.001
RPG (mmol/L)	5.64 ± 2.19	6.00 ± 2.57	6.55 ± 2.54	6.54 ± 2.31	<0.001
FPG (mmol/L)	5.50 ± 1.37	5.78 ± 1.61	6.04 ± 1.86	6.22 ± 1.78	<0.001
OGTT (mmol/L)	5.95 ± 2.22	6.30 ± 2.64	6.68 ± 2.96	7.01 ± 2.81	<0.001
HbA1c (%)	5.43 ± 0.78	5.56 ± 0.88	5.66 ± 0.97	5.77 ± 1.01	<0.001
TC (mg/dL)	199.55 ± 38.81	193.68 ± 40.38	192.94 ± 41.72	192.88 ± 44.41	<0.001
TG (mg/dL)	106.89 ± 44.02	118.00 ± 68.44	131.49 ± 65.49	158.30 ± 107.19	<0.001
LDL-C (mg/dL)	112.30 ± 24.90	114.91 ± 25.74	115.30 ± 26.18	114.65 ± 25.19	<0.001
UA (mg/dL)	4.08 ± 0.85	5.04 ± 0.89	5.78 ± 0.92	6.84 ± 1.19	<0.001
HDL-C (mg/dL)	71.56 ± 16.13	56.32 ± 10.14	47.63 ± 7.83	37.87 ± 7.18	<0.001
Non-HDL-C (mg/dL)	127.99 ± 36.84	137.37 ± 39.88	145.32 ± 41.40	155.01 ± 44.38	<0.001
Male (%)	14.40	38.77	62.23	79.54	<0.001
Race (%)					<0.001
Mexican American	7.05	8.98	9.22	8.93	
Other Hispanic	5.14	5.59	5.06	5.53	
Non-Hispanic White	69.06	66.53	67.79	69.15	
Non-Hispanic Black	11.57	11.99	10.46	8.97	
Other Race	7.20	6.90	7.48	7.42	
Education (%)					<0.001
Less than high school	13.59	17.94	17.73	19.31	
High school or GED	18.84	22.41	22.80	25.83	
College or above	67.57	59.64	59.47	54.86	
PIR (%)					<0.001
<1.30	17.61	21.19	20.33	21.45	
1.30-3.49	38.63	39.91	40.31	41.45	
≥3.50	43.77	38.90	39.36	37.09	
Drinking (%)	11.53	11.56	10.73	10.27	0.117
Smoking (%)	39.69	42.17	48.35	51.12	<0.001
Hypertension (%)	22.34	28.49	35.04	40.91	<0.001
Diabetes (%)	6.63	10.88	14.15	18.02	<0.001

Values for categorical variables are reported as weighted percentages; for continuous variables, as weighted mean ± standard deviation. Statistical analysis included Weighted Analysis of Variance (ANOVA) for continuous variables and chi-squared test for categorical variables.

**Figure 2:**
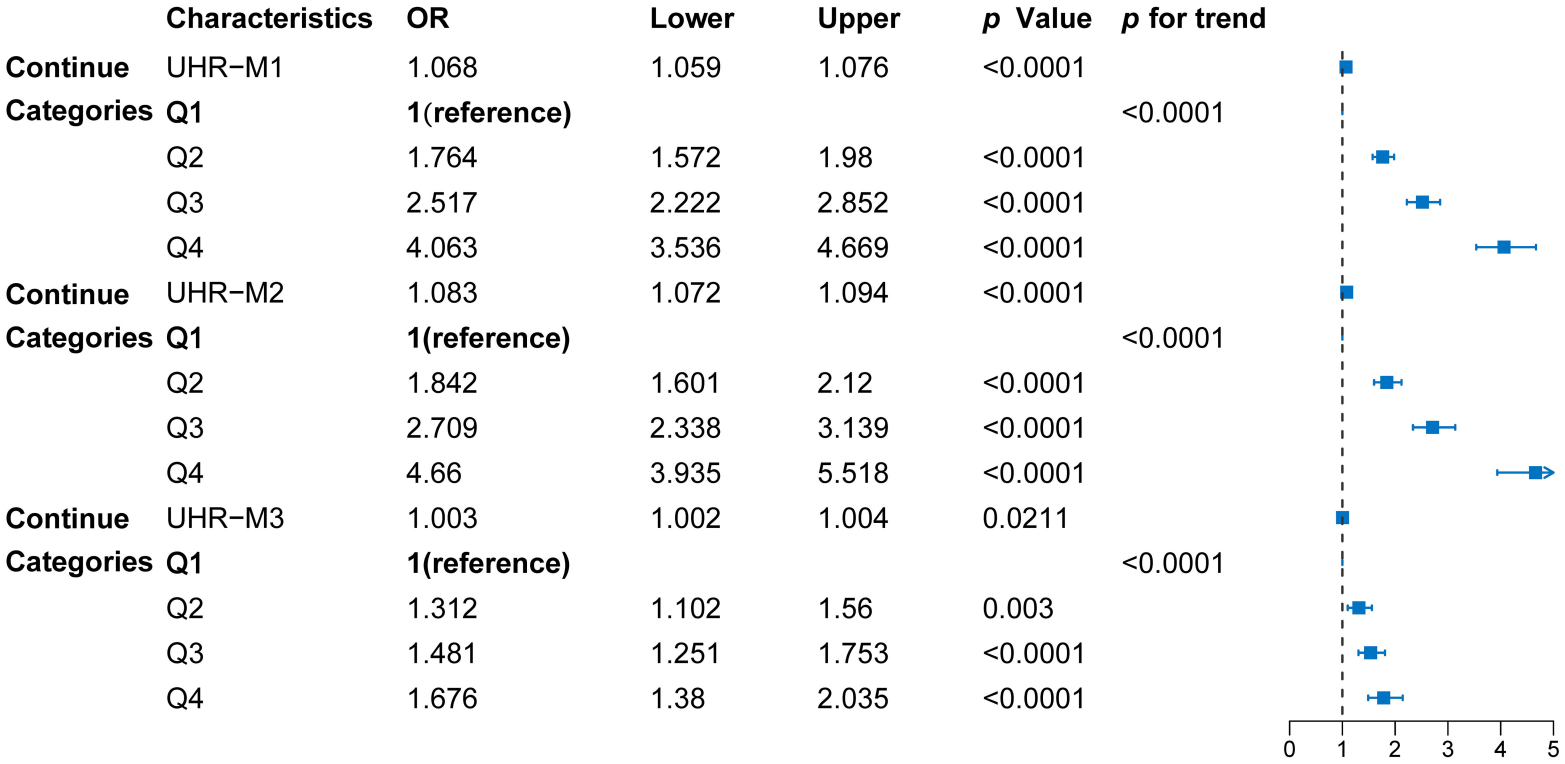
Forest diagram of the association between UHR quartiles and diabetes risk. Model 1: unadjusted; Model 2: adjusted for sex, age, and race; Model 3: adjusted for sex, age, race, BMI, PIR, hypertension, smoking and drinking status, SBP, DBP, TC, TG, LDL-C, and non-HDL-C. Data points represent odds ratios (OR) with 95% confidence intervals (CI), derived from multivariable logistic regression models.

Further multivariate logistic regression models were constructed to assess the independent association between UHR and diabetes (see **Figure 2**). Variance inflation factor (VIF) analysis showed that all variables had GVIF values below 2, well within the common collinearity threshold of 10, indicating no severe multicollinearity issues among the covariates. Thus, the coefficients in the regression model are stable and interpretable, unaffected by multicollinearity. The unadjusted model (Model 1) indicated that UHR was significantly associated with diabetes (*p*<0.001); participants in the highest quartile (Q4) had a fourfold increased risk of diabetes compared to those in the lowest quartile (Q1) (OR = 4.063, 95% CI: 3.536–4.669, *p* < 0.001). After adjusting for age, sex, ethnicity, and other factors (Model 2), the association between UHR and diabetes remained significant (*p*<0.001). Even with additional adjustments for metabolic and lifestyle variables like BMI, TC, TG, LDL-C and Non-HDL-C (Model 3), UHR was still significantly associated with diabetes risk (*p*<0.001). These findings suggest that higher UHR is positively associated with increased diabetes risk, and this association remains significant even after adjusting for various confounding factors.

### 3.3 Subgroup analysis: predictive value of UHR in different populations

**Figure 3:**
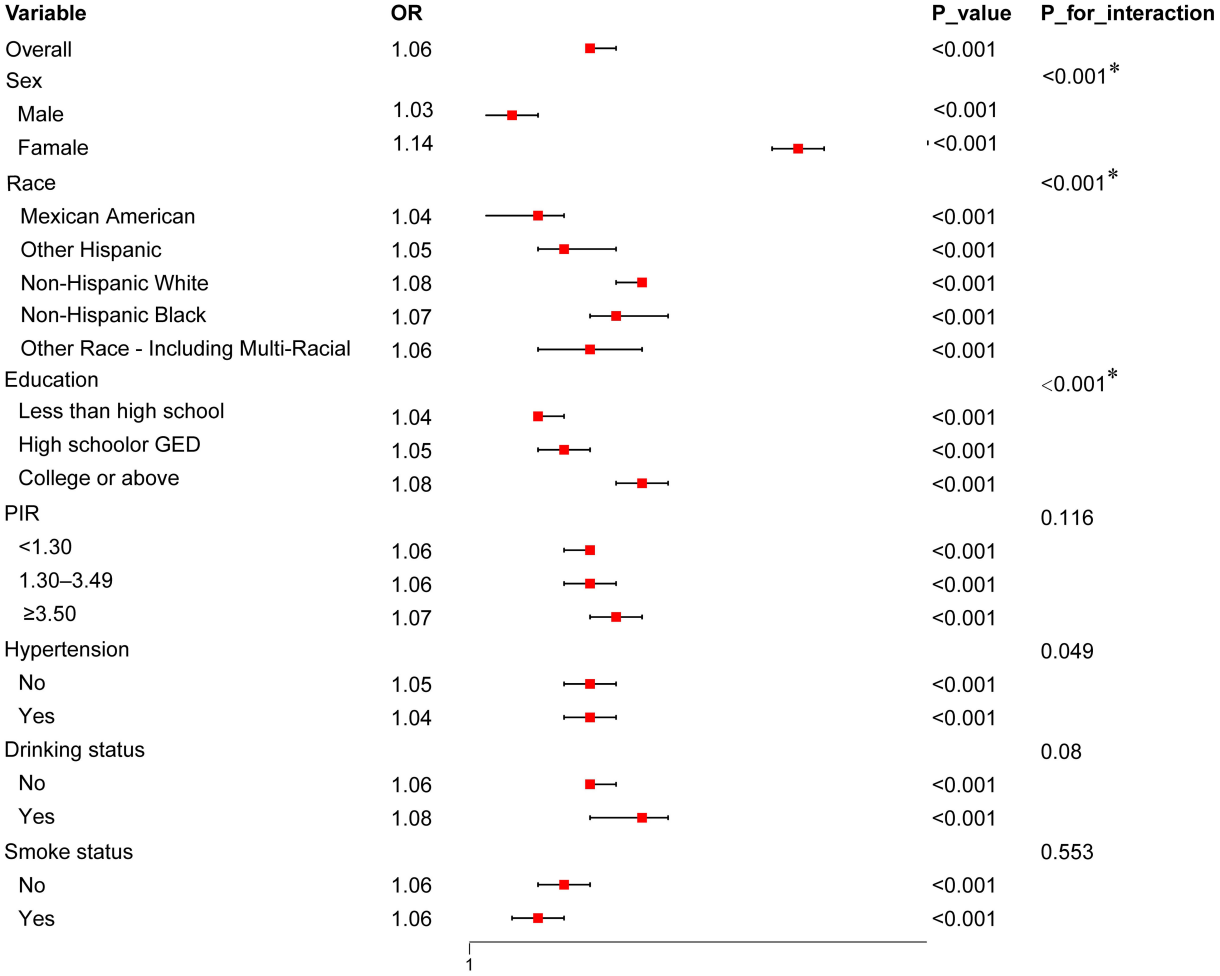
Forest Plot of Subgroup Analysis for the association between UHR and diabetes risk. The plot presents OR with 95% CI across various subgroups, including sex, race, education level, PIR, hypertension status, drinking status, and smoking status. Interaction p-values (P_for_interaction) indicate whether there is a statistically significant difference in the association across subgroups. Only values with *p < 0.00714 (adjusted for multiple comparisons using Bonferroni correction) are considered statistically significant.

To further investigate the predictive value of UHR for diabetes risk across different populations, we performed subgroup analyses based on factors such as sex, ethnicity, and education level (see **Figure 3**). The results indicated that the predictive value of UHR was approximately 11% higher in females than in males, with OR values of 1.14 and 1.03, respectively, and the interaction effect was significant (*p*<0.001). In the racial subgroup analysis, the predictive effect of UHR was slightly higher in non-Hispanic whites compared to other racial groups. Moreover, the predictive value of UHR was enhanced among participants with higher education levels. However, UHR did not exhibit a significant predictive effect in subgroups stratified by PIR, hypertension, smoking and drinking status.

### 3.4 Variable selection via LASSO regression

**Figure 4:**
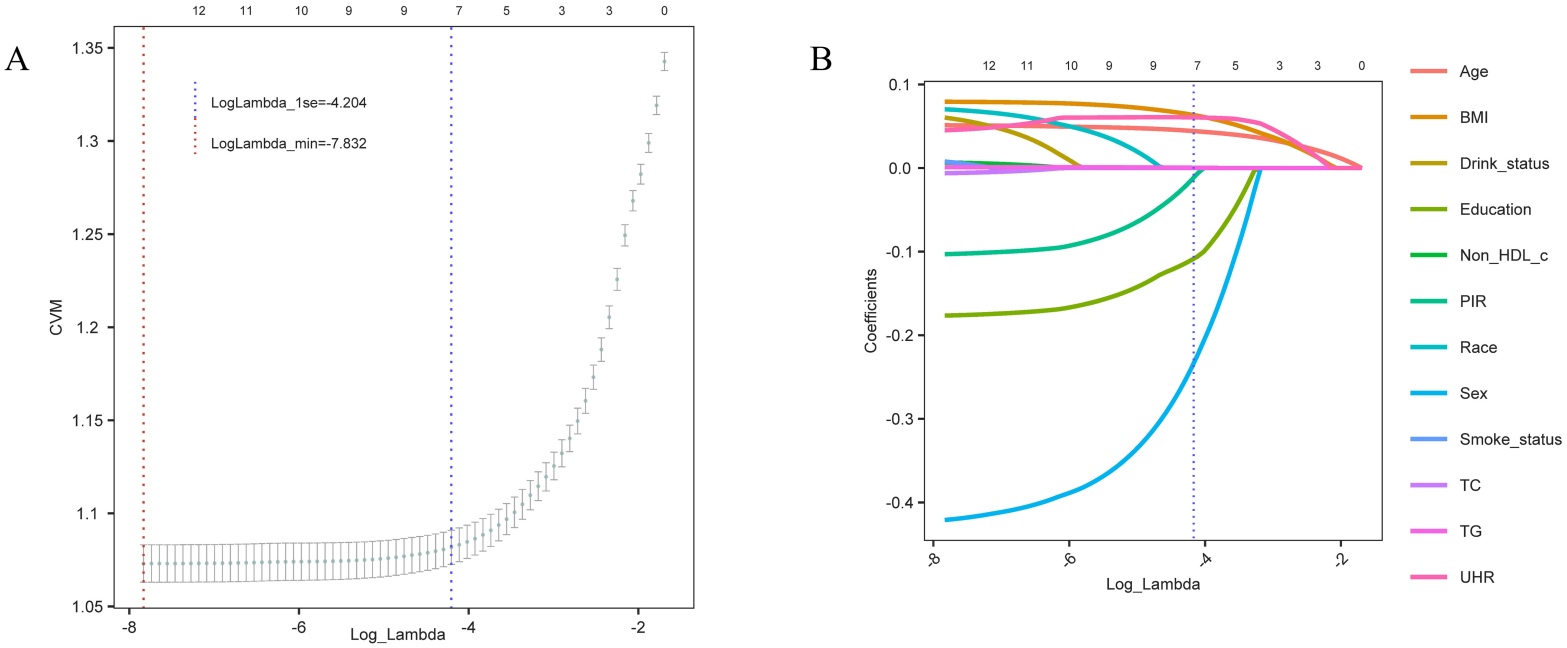
Variable selection trajectory of the LASSO regression model. **(A)** Cross-validation error (CVM) plot for different Log_Lambda values, with the red dashed line indicating the minimum error (LogLambda_min) and the blue dashed line showing the one-standard-error threshold (LogLambda_1se) for model selection. **(B)** Coefficient trajectories for each variable as Log_Lambda varies. The blue dashed line represents the selected Log_Lambda (LogLambda_1se), indicating the key predictive variables retained in the final model.

To build a more concise and efficient predictive model, we applied LASSO regression to select the variables included (**Figure 4**). The optimal lambda value was determined via cross-validation (LogLambda_1se = -4.204, **Figure 4A**), leading to the selection of key variables such as UHR, BMI, age, sex, and education level (**Figure 4B**). LASSO regression aids in simplifying the model and preventing overfitting, further validating the independent role of UHR as a predictive factor for diabetes risk.

### 3.5 Multivariate logistic regression models and model evaluation

**Figure 5:**
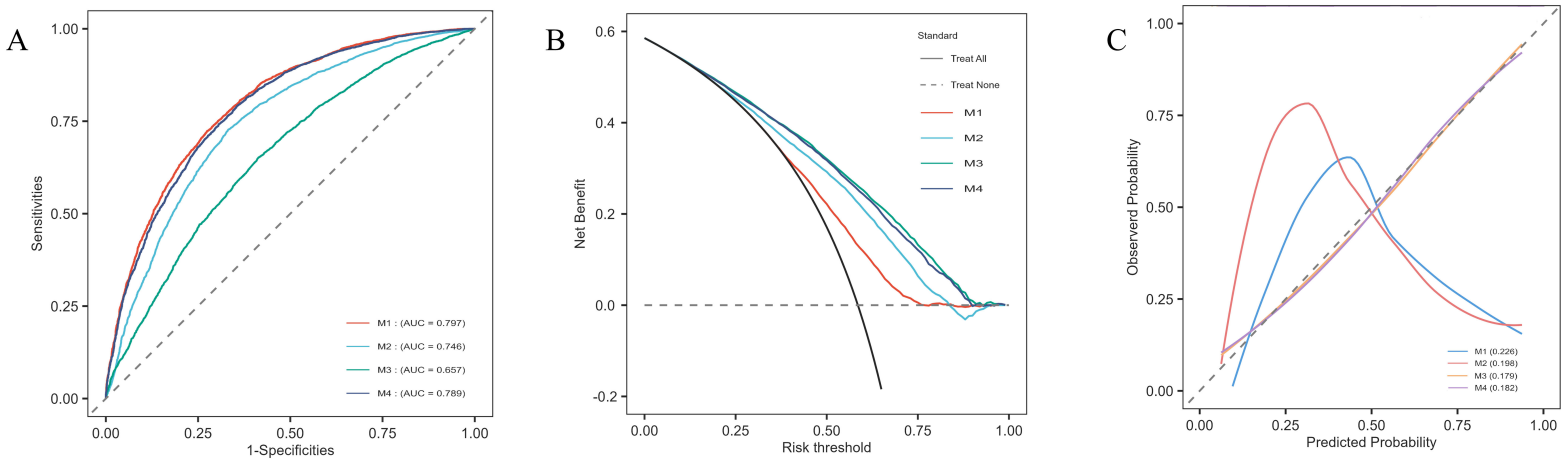
Model evaluation plots for the multivariate logistic regression models. **(A)** ROC curves for each model, showing the sensitivity and specificity of UHR in predicting diabetes risk, with corresponding AUC values: M1 (0.797), M2 (0.746), M3 (0.657), and M4 (0.789). **(B)** DCA displaying net benefit across different risk thresholds, comparing the four models. **(C)** Calibration curves illustrating the agreement between predicted and observed probabilities for each model, demonstrating the calibration accuracy.

Using the variables selected via LASSO regression, we constructed multivariate logistic regression models and assessed their predictive capabilities through ROC curves, DCA, and calibration curves. The results indicated that the model including UHR (M1) had a high AUC value (AUC=0.797), suggesting that UHR alone demonstrated good predictive ability for diabetes risk (**Figure 5A**). The LASSO-selected model (M4) had an AUC of 0.789; although slightly lower than M1, the model exhibited better stability and overall predictive performance. Particularly in the DCA, M4 demonstrated higher net benefits (**Figure 5B**), and the calibration curve showed good concordance between predicted results and actual observations (**Figure 5C**). Additionally, the Hosmer-Lemeshow test results showed that the p-values for all models were greater than 0.05, indicating good model fit with no significant differences between predicted and observed values.

**Figure 6:**
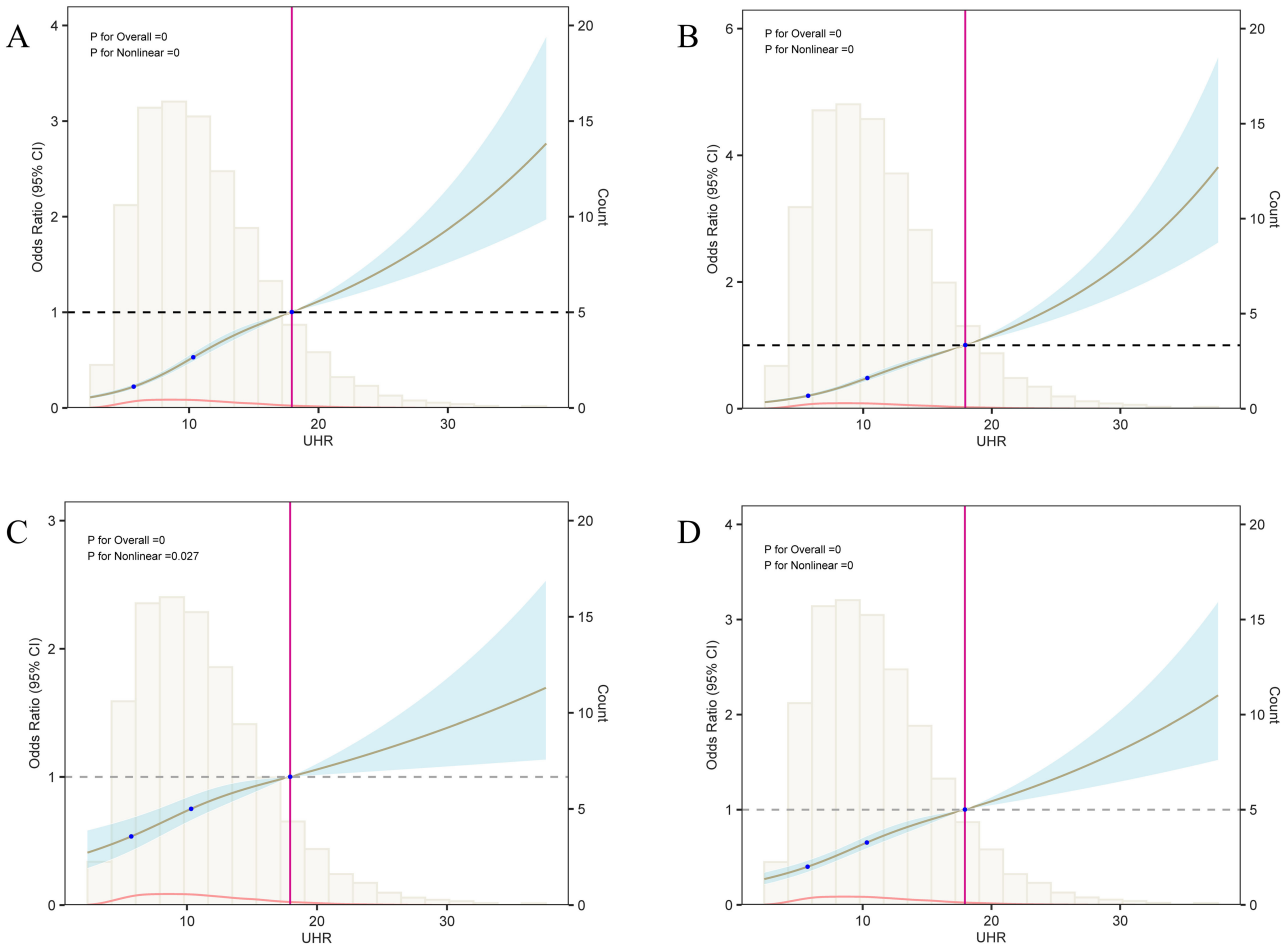
Analysis of the nonlinear relationship between UHR and diabetes risk. This figure presents the results of the restricted cubic spline (RCS) analysis between UHR and diabetes risk; panels **(A–D)** correspond to the RCS curves of models 1 through 4, respectively. Each model’s three knot positions are located at 5.67, 10.37, and 18. When UHR values exceed 18, the odds ratio (OR) surpasses 1, suggesting a significant increase in diabetes risk. Threshold analysis revealed that the UHR inflection points for models 1 to 4 are 10.19, 12.27, 12.29, and 10.00, respectively, indicating that after these inflection points, the effect of UHR on diabetes risk progressively strengthens.

Additionally, the nonlinear association between UHR and diabetes risk was confirmed across different models (**Figure 6**). In the M1 model containing only UHR (**Figure 6A**), diabetes risk increased progressively as UHR increased. Threshold analysis revealed an inflection point at 10.19 in the model, indicating that when UHR reaches 10.19, although the OR value is still less than 1, the rate of risk increase begins to accelerate; when UHR exceeds 18, the OR value surpasses 1, and the diabetes risk increases significantly. Even after adjusting for age, sex, ethnicity (M2, **Figure 6B**), and further including metabolic indicators like BMI, TC, TG, LDL-C, Non-HDL-C, and PIR (M3, **Figure 6C**), the nonlinear association between UHR and diabetes remained significant, with inflection points at 12.27 and 12.29, respectively. In the M4 model, incorporating variables selected via LASSO, the nonlinear association of UHR persisted with an inflection point at 10.00 (**Figure 6D**), further confirming UHR’s role as an independent predictive marker. The existence of the inflection point suggests that when UHR exceeds 10.00, the rate of risk increase accelerates; when UHR surpasses 18, the risk increases markedly. This offers explicit reference points for clinical intervention.

**Figure 7:**
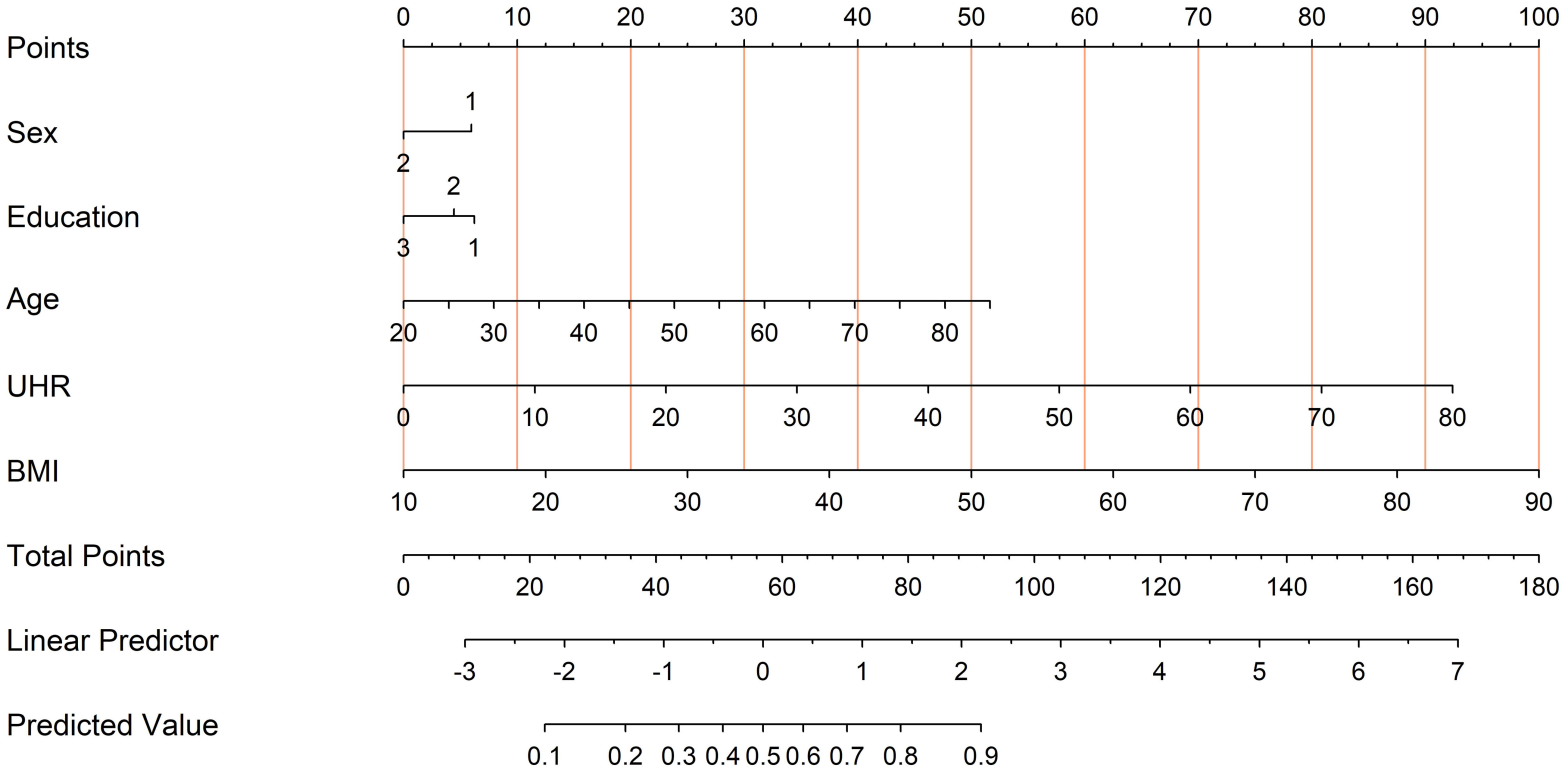
Nomogram derived from the multivariate logistic regression model for diabetes risk prediction. To use the nomogram, locate each predictor’s value (Sex, Education, Age, UHR, and BMI) on its respective axis and draw a line up to the “Points” scale to assign points. Sum all points and find the total on the “Total Points” line. This total corresponds to the predicted probability of diabetes at the bottom of the nomogram.

Finally, a nomogram based on multivariate logistic regression was developed to provide a practical tool for individualized diabetes risk assessment (**Figure 7**). This model combines UHR, age, BMI, sex, and education level, assisting in providing quantitative references for personalized diabetes risk prediction.

## 4 Discussion

While a few studies have preliminarily explored the relationship between UHR and diabetes (15, 16), comprehensive studies in large-scale populations remain relatively scarce. By analyzing the NHANES 2005-2018 database, this study included 30,813 participants to assess the independence of UHR as a risk predictor for diabetes. We initially performed univariate, multivariate, and subgroup analyses, followed by constructing multivariate logistic regression models. The models’ predictive capabilities were assessed through ROC curves, DCA, and calibration curves. These methods revealed a significant nonlinear relationship between UHR and diabetes risk. Even after accounting for various confounding factors, UHR remained an independent risk indicator for diabetes. The results suggest that UHR may hold potential value for early diabetes risk assessment.

This study found that UHR levels were significantly higher in the diabetes group than in the non-diabetes group, and diabetes prevalence increased markedly with rising UHR levels. Further logistic regression analysis showed that in the unadjusted Model 1, each unit increase in UHR was associated with a 6.8% increase in diabetes risk; individuals in the highest quartile had four times the risk of diabetes compared to those in the lowest quartile. This association remained significant in the adjusted Model 2 and the fully adjusted Model 3, confirming the independence of UHR as a predictor of diabetes risk. These findings align with previous studies, which have demonstrated a close association between UHR and diabetes as well as related metabolic diseases (20, 21). This association may stem from the components of UHR—UA and HDL-C. As the end product of purine metabolism, UA has been shown to be closely related to insulin resistance, oxidative stress, and inflammation (24, 25); elevated UA levels can promote oxidative stress and inflammatory responses, increasing the risk of insulin resistance (26, 27). In contrast, HDL-C exerts anti-inflammatory and antioxidant effects, and its decrease often signifies worsening metabolic dysfunction, which in turn raises the risk of diabetes (28). Therefore, UHR as a composite indicator may reflect the cumulative effects of multiple metabolic abnormalities and serve as an important marker for diabetes and other metabolic diseases.

Additionally, studies have shown that when UHR surpasses a specific threshold, it exhibits significantly higher sensitivity and specificity in prediabetes screening (16). In this study, restricted cubic spline (RCS) analysis demonstrated a significant nonlinear relationship between UHR and diabetes risk, with an approximate threshold at UHR = 10. This suggests that once UHR exceeds this level, the risk of diabetes rises sharply. Such a threshold effect may indicate multiple pathological mechanisms triggered by elevated UHR, including increased oxidative stress, endothelial dysfunction, and systemic inflammation. For instance, excessively high uric acid levels can impair vascular function through oxidative stress and inflammatory responses, while reduced HDL-C levels diminish antioxidant and anti-inflammatory defenses, further exacerbating insulin resistance (29). Similar nonlinear effects have been observed in other metabolic-related diseases, where biomarkers such as waist circumference or blood pressure, once beyond certain thresholds, lead to a marked increase in the risk of metabolic disorders and cardiovascular events, particularly in high-risk individuals (30, 31). Future research should explore the mechanisms underlying the threshold effect of UHR and validate these findings with prospective data to better identify high-risk populations in clinical settings.

This study also evaluated the predictive performance of UHR for diabetes through ROC curves and DCA, showing that UHR has strong predictive ability for diabetes and further confirming its independence as a risk predictor. A nomogram constructed with covariates selected via LASSO regression, incorporating UHR alongside other non-invasive factors (such as sex, age, and BMI), further enhanced its utility in diabetes risk prediction. Compared to single metabolic markers, UHR, as a composite indicator, offers a more comprehensive reflection of complex metabolic disturbances, giving it a unique advantage in risk prediction (32). Previous studies have shown that UHR provides additional metabolic insights beyond traditional diabetes predictors, especially in individuals with multiple metabolic abnormalities (33). Moreover, UHR’s effectiveness has been validated in metabolic diseases like NAFLD and MAFLD, highlighting its potential as a non-invasive screening tool (19, 34). The nomogram developed in this study may serve as a reference for diabetes risk screening in clinical settings and support future efforts to optimize UHR’s predictive performance through multivariable models.

Additionally, subgroup analysis showed that the association between UHR and diabetes risk was significant and consistent across all subgroups, but with stronger predictive effects observed among females, non-Hispanic whites, and individuals with higher education levels. This variation may be related to the metabolic characteristics, lifestyle, or socioeconomic status of each subgroup. For instance, female hormones like estrogen have anti-inflammatory effects and regulate lipid metabolism, which may influence uric acid and HDL-C levels, making UHR a more sensitive predictor of diabetes risk in women (35, 36). Interestingly, a study found that UHR exhibited a stronger predictive value for metabolic syndrome among men than women in non-diabetic populations (17). This suggests that gender differences may impact UHR’s predictive efficacy in various diseases, though systematic studies exploring these differences in diabetic populations remain limited; future research could investigate the underlying reasons for such gender-specific effects. Additionally, differences in lifestyle and genetic factors among non-Hispanic whites may contribute to a more pronounced association between UHR and diabetes risk in this group. Some studies have indicated that non-Hispanic whites exhibit distinct genetic susceptibility to metabolic disorders compared to other ethnicities (37). However, due to the internal diversity within Hispanic populations, with varying health outcomes among subgroups, further validation is needed to confirm UHR’s predictive role for diabetes in non-Hispanic whites. Among individuals with higher education levels, generally healthier lifestyles tend to lower overall health risks, contributing to more stable metabolic indicators (38). However, our findings suggest that elevated UHR in higher-educated groups may serve as a more sensitive indicator of underlying diabetes risk, although whether this sensitivity holds across other educational levels requires further investigation.

Although our subgroup analysis showed that UHR did not exhibit significant predictive effects in groups based on PIR, hypertension, smoking, and drinking status, previous studies suggest that UHR may still hold predictive value within these populations. For instance, one study found a positive correlation between serum UHR levels and hypertension among women of reproductive age, suggesting that UHR could be a potential clinical marker for hypertension (39). Thus, our findings do not rule out the potential of UHR as a predictive marker in hypertensive or other specific populations. Furthermore, PIR primarily reflects socioeconomic status, while smoking and drinking behaviors reflect lifestyle habits, which can vary considerably across different social groups or disease states and may influence the predictive performance of UHR. It should also be noted that these variables were collected through self-reported questionnaires, which may introduce recall bias and affect the results. Overall, UHR’s role may vary in complexity and significance across different populations, warranting further exploration. Future research should aim to investigate UHR’s potential as a metabolic or disease risk predictor in broader populations and under more precise classification standards.

This study utilized large-scale, nationally representative data from the NHANES 2005-2018, which enhances the generalizability of the findings. Through multivariate analysis, subgroup analysis, and multivariate logistic regression models, we effectively controlled for several confounding factors. Non-invasive variables such as sex, age, BMI, and education level were selected using LASSO regression, further improving UHR’s predictive ability for diabetes risk. Subsequently, we evaluated the model’s predictive performance using ROC curves, DCA, and calibration curves, and ultimately visualized UHR’s potential clinical utility in predicting diabetes risk through a nomogram. However, this study also has several limitations. First, as this study is based on cross-sectional NHANES data, we cannot establish a causal relationship between UHR and diabetes risk, and there is a lack of long-term follow-up data to evaluate the long-term predictive effects of UHR. Future research should employ longitudinal cohort studies, such as the approach used by Cai et al., using Cox regression and survival analysis to further verify the role and stability of UHR in predicting long-term diabetes risk (40, 41). Second, due to the significant missing data for variables such as physical activity and dietary patterns in the NHANES 2005-2018 cycles, these factors were not included in the analysis. Future studies should consider shortening the study period, selecting more complete datasets, or adopting effective data imputation methods to more comprehensively assess the impact of these variables on the relationship between UHR and diabetes. Finally, while this study explored the impact of UHR across different populations through subgroup analysis, future research should delve deeper into gender differences and other subgroup mechanisms, and combine mediation analysis to clarify the potential mechanisms through which UHR influences diabetes risk. Additionally, *in vitro* and *in vivo* studies could further validate the biological mechanisms underlying UHR’s predictive role, exploring its effects on metabolic and inflammatory pathways to provide stronger foundational evidence for clinical applications.

## 5 Conclusion

This study suggests that UHR may be an independent predictor of diabetes risk, with the risk of diabetes increasing significantly as UHR levels rise. Multivariate regression, ROC curve, and nonlinear analyses all demonstrated that UHR remains a significant predictor of diabetes risk even after adjusting for various confounding factors. Subgroup analysis revealed differences in UHR’s predictive effect across different populations, with a particularly stronger effect observed in females. Given that UHR is a simple and readily accessible biomarker, its potential application in early diabetes screening warrants further validation and investigation.

## Data Availability

Publicly available datasets were analyzed in this study. This data can be found here: https://wwwn.cdc.gov/nchs/nhanes/Default.aspx.
